# Characterization of *Salmonella enterica* Contamination in Pork and Poultry Meat from São Paulo/Brazil: Serotypes, Genotypes and Antimicrobial Resistance Profiles

**DOI:** 10.3390/pathogens11030358

**Published:** 2022-03-16

**Authors:** Vasco T. M. Gomes, Luisa Z. Moreno, Ana Paula S. Silva, Siddhartha Thakur, Roberto M. La Ragione, Alison E. Mather, Andrea M. Moreno

**Affiliations:** 1 Department of Preventive Veterinary Medicine and Animal Health, School of Veterinary Medicine and Animal Science, University of São Paulo, Av. Prof. Dr. Orlando Marques de Paiva, 87, São Paulo 05508-270, Brazil; gomesvtm@gmail.com (V.T.M.G.); luzanolli@gmail.com (L.Z.M.); anapaula_silva2006@yahoo.com.br (A.P.S.S.); 2 Department of Population Health and Pathobiology, College of Veterinary Medicine, North Carolina State University, Raleigh, NC 27606, USA; sthakur@ncsu.edu; 3 Department of Pathology and Infectious Diseases, School of Veterinary Medicine, Faculty of Health and Medical Sciences, University of Surrey, Guildford GU2 7AL, UK; r.laragione@surrey.ac.uk; 4 Quadram Institute Bioscience, Norwich NR4 7UQ, UK; alison.mather@quadram.ac.uk; 5 Faculty of Medicine and Health Sciences, University of East Anglia, Norwich NR4 7TJ, UK

**Keywords:** *Salmonella enterica*, pork, poultry, serotype, antimicrobial resistance, PFGE

## Abstract

Salmonellosis is a zoonosis of major relevance to global public health. Here we present the assessment of *Salmonella enterica* contamination in pork and poultry meat sold at retail markets in São Paulo, Brazil. A total of 780 meat samples (386 poultry meat and 394 pork samples) were collected from 132 markets. From these, 57 samples (7.3%) were positive for *S. enterica* isolation, including 32 (8.3%) poultry meat and 25 (6.3%) pork samples. *S. enterica* isolates were further characterized for serotyping, antimicrobial resistance and genotyping by amplified fragment length polymorphism and pulsed field gel electrophoresis. Antimicrobial resistance analysis demonstrated two main profiles: pork isolates were more resistant to macrolides, β-lactams, tetracycline, phenicols, and fluoroquinolones, and poultry meat isolates presented higher resistance to fluoroquinolones, sulfonamides, tetracycline, and β-lactams. A total of 72.4% of poultry meat isolates were identified as *S.* Heidelberg, while most of pork isolates were *S.* Typhimurium (31.7%) and *S*. Give (16.7%). Genotyping resulted in most clusters consisting exclusively of pork or poultry meat, no cross-contamination was detected, and a tendency to differentiate isolates according to their serotypes and markets of origin. High resistance rates to critically important antimicrobials reinforce the importance of controlling Salmonella contamination in meat production chains.

## 1. Introduction

Non-typhoidal salmonellosis is a major zoonosis with relevance to global public health. According to the World Health Organization (WHO), each year, approximately 1 in 10 people become ill with foodborne infections and 33 million years of healthy life are lost. Diarrheal diseases are the most common result of foodborne infections and approximately 550 million people fall ill each year. Salmonella infection is considered one of the four leading global causes of diarrheal diseases [1]. However, few countries report complete data on the population and economic impacts caused by salmonellosis [2,3].

In the United States, the Centers for Disease Control and Prevention (CDC) associates *Salmonella* infection with 1.35 million cases of foodborne disease, 26,500 hospitalizations, and 420 deaths annually [4]. Although the overall incidence has decreased in recent years, an increase in infections caused by serotypes Infantis, Muenchen, Montevideo, and Braenderup was detected [5]. In Europe, salmonellosis was the second most frequent foodborne disease, with 87,923 confirmed human cases in 2019; serovars *S.* Enteritidis (50.3%), *S*. Typhimurium (11.9%), and monophasic *S*. Typhimurium (8.2%) were the most reported among cases with known serovar [6].

In Brazil, *S. enterica* has been reported as one of the main pathogens associated with foodborne disease over the last 15 years and has been associated with approximately 34.1% of the outbreaks reported between 2000 and 2017 [7]. However, the incompleteness of these data due to underreporting of the occurrences of gastrointestinal conditions without hospitalization need should be highlighted. Furthermore, the official reports in Brazil do not provide the identification of Salmonella serovars. Nevertheless, according to Campioni et al. [8], *S.* Enteritidis was the most prevalent serovar isolated from Brazilian foodborne outbreaks from the late 1980s to early 2000s.

Although *Salmonella* infection has been associated with the consumption of varied foods, animal products play a prominent role as an infection source [9,10]. Meat, especially pork and poultry meat, has been traditionally involved in the dissemination of *Salmonella* to humans and plays an important role in the epidemiology of distinct serovars [11]. However, most of the recent Brazilian studies and monitoring were performed at the beginning of the production chain, mainly farms and slaughterhouses, focusing on carcass contamination [12,13,14,15], but not retail meat. Therefore, further evaluation of retail meat contamination was required considering the direct risks to consumers. The aim of this study was to assess the *Salmonella enterica* contamination in pork and poultry retail meat sold in distinct market types in São Paulo city (Brazil), including genotyping and antimicrobial resistance profiling of the obtained isolates.

## 2. Materials and Methods

### 2.1. Sampling

A total of 17 sampling trips were performed between September 2013 to April 2016, covering 132 markets from the 5 macro-regions of São Paulo city. These included hypermarkets (10.6%), supermarkets (12.1%), neighborhood butchers (46.2%), and butcher stalls within municipal markets (31.1%). The evaluated markets were also classified if they sold exclusively poultry meat (15.9%) or pork (25.0%), or if both pork and poultry meat were handled and commercialized (59.1%). A total of 780 meat samples were evaluated—394 pork and 386 poultry meat samples—including different meat cuts: chop (97), gammon (93), loin (101), rib (103) among pork samples, and wing (92), thigh (97), breast (98), and drumstick (99), among poultry meat samples.

### 2.2. Salmonella Isolation and Confirmation

The *S. enterica* isolation was performed according to Holt et al. [16]. Briefly, 25 g of each sample were homogenized in 225 mL of sterile buffered peptone water with 4% novobiocin and incubated at 37 °C for 24 h, aerobically. From this pre-enrichment broth, 1.0 mL was transferred to 9 mL of tetrathionate added with iodine solution broth (Difco, Detroit, MI, USA) and incubated at 37 °C for 24 h, aerobically. One loopful of the enrichment broth was plated onto Xylose Lysine Tergitol 4 agar (XLT4, Difco), CHROMagar^®^ Salmonella (Difco) and MacConkey agar (Difco) and incubated at 37 °C for 24 to 48 h, aerobically. Two to six presumptive *Salmonella* colonies from each sample were selected for further analyses.

The selected *S. enterica* colonies were confirmed by Matrix Associated Laser Desorption-Ionization—Time of flight mass spectrometry–MALDI-TOF (Bruker Daltonics, Inc. Billerica, MA, USA) and invA gene amplification. For MALDI-TOF MS identification, sample preparation and processing were performed according to Hijazin et al. [17]. A Microflex^®^ mass spectrometer (Bruker Daltonics, Inc. Billerica, MA, USA) was used for mass spectra acquisition in the 2–20 kDa range. The obtained spectra were loaded into MALDI BioTyper^®^ 3.0 and compared with the manufacturer’s library; standard Bruker interpretative criteria were applied for microbial identification. For molecular *S. enterica* identification, purified DNA was recovered using the protocol by Boom et al. [18], and the partial amplification of *invA* gene was performed as previously described by Rahn et al. [19].

### 2.3. Serotyping

The antigenic characterization of *S. enterica* was obtained using the fast agglutination technique based on the antigenic formulas for *Salmonella* [20].

### 2.4. Antimicrobial Resistance Profiling

For the evaluation of antimicrobial resistance, the broth microdilution technique was applied, according to the CLSI VET08 [21] standards, to determine the minimum inhibitory concentrations (MICs) using a 17 antimicrobials panel. The Staphylococcus aureus ATCC 29,213 was used as quality control strain. The obtained MIC results were categorized as susceptible, intermediate, and resistant using the interpretative criteria specified in CLSI performance standards VET08 [21] and M100 [22]. The multidrug resistance (resistance to three or more classes of antimicrobials) rate was determined as described by Schwarz et al. [23].

### 2.5. Genotyping

For single-enzyme amplified fragments length polymorphism (SE-AFLP), DNA was recovered according to the extraction protocol by Boom et al. [18]. The AFLP was performed as previously described by McLauchlin et al. [24], using the restriction endonuclease HindIII (New England BioLabs Inc., Ipswich, MA, USA). The amplified products were detected with electrophoresis at 90 V for 4 h in 2% agarose gel stained with BlueGreen^®^ (LGC Biotecnologia, São Paulo, Brazil) and photographed under UV transillumination system Gel Doc XR^®^ (Bio-Rad Laboratories, Hercules, CA, USA). The 100 pb DNA Ladder (New England BioLabs Inc., Ipswich, MA, USA) was applied for amplified fragments determination.

The Pulsed Field Gel Electrophoresis (PFGE) culture conditions, plug preparation, and DNA extraction were performed using Ribot et al. [25] and Pulsenet 2017 protocol. The XbaI (New England BioLabs Inc.) restriction enzyme was applied for DNA digestion at 37 °C for 2 h. Electrophoresis was performed using 1% SeaKem Gold agarose (Cambrex Bio Science Rockland, Inc., East Rutherford, NJ, USA) and a CHEF-DR III System (Bio-Rad Laboratories) with 0.5× TBE at 14 °C. DNA fragments were separated in the following conditions: run time 20 h at 6 V/cm at 120° fixed angle with pulse times from 2.2 to 63.8 s. Finally, gels were stained with 1× SYBR^®^ Safe (Invitrogen Corporation, Carlsbad, CA, USA) for 30 min and visualized under UV transillumination system Gel Doc XR^®^ (Bio-Rad Laboratories). Lambda DNA-PFGE^®^ marker (New England BioLabs Inc., Ipswich, MA, USA) and *Salmonella* serotype Braenderup H9812 were applied as standard and for fragment size determination.

### 2.6. Statistical Analysis

The descriptive analyses were performed using SPSS 16.0 (SPSS Inc, Chicago, IL, USA). The resistance results were transformed into binary data for identification of the respective resistance profiles and subsequent cluster analysis. Profiles were analyzed as categorical data in Bio Numerics 7.6 (Applied Maths, Sint-Martens-Latem, Belgium), and a dendrogram was constructed using the different values coefficient and Ward method.

The SE-AFLP and PFGE fingerprint patterns were analyzed with BioNumerics 7.6 (Applied Maths, Sint-Martens-Latem, Belgium) to generate dendrogram using the Dice coefficient and UPGMA (unweighted pair group method with arithmetic mean) method. For SE-AFLP analysis, a 90% genetic similarity cut-off value was applied to analyze the resulting clusters; for the PFGE cluster analysis, the isolates were considered in different pulsotypes when they differed by four or more bands [26]. The respective discriminatory indexes were calculated according to Hunter and Gaston [27].

## 3. Results

From the 780 analyzed meat samples (386 poultry meat and 394 pork samples), only 57 samples (7.3%) were positive for *Salmonella enterica*, including 32 (32/386–8.3%) poultry meat samples and 25 (25/394–6.3%) pork samples (Table 1). These originated from 35 markets, of which 57.1% were classified as neighborhood butchers, 25.7% as supermarkets, 11.4% as municipal markets, and 5.7% as hypermarkets. Among positive markets, 82.9% sold both pork and poultry meat. However, *S. enterica* was only isolated simultaneously from both pork and poultry in samples obtained from four of the studied markets (M85, M88, M99, and M129).

From the 57 positive meat samples, 32 poultry meat and 25 pork samples, 58 isolates from poultry meat, and 60 isolates from pork were further selected for serotyping, genotypic analysis, and antimicrobial resistance profiling. The selection included one to three strains identified as *Salmonella enterica* from each positive sample.

A total of 12 different serotypes were detected among studied isolates (Table 2). Only serotype Schwarzengrund appeared in both pork and poultry meat; Heidelberg (72.4%) was the most frequent among poultry meat isolates, while Typhimurium (31.7%) and Give (16.7%) were more prevalent in pork. It is highlighted that only five markets (M39, M85, M99, M113, and M129) presented more than one serotype among the tested meat samples, of which M39 and M113 had only one positive sample each with two distinct serotypes detected among their respective isolates (Table S1).

**Figure 1:**
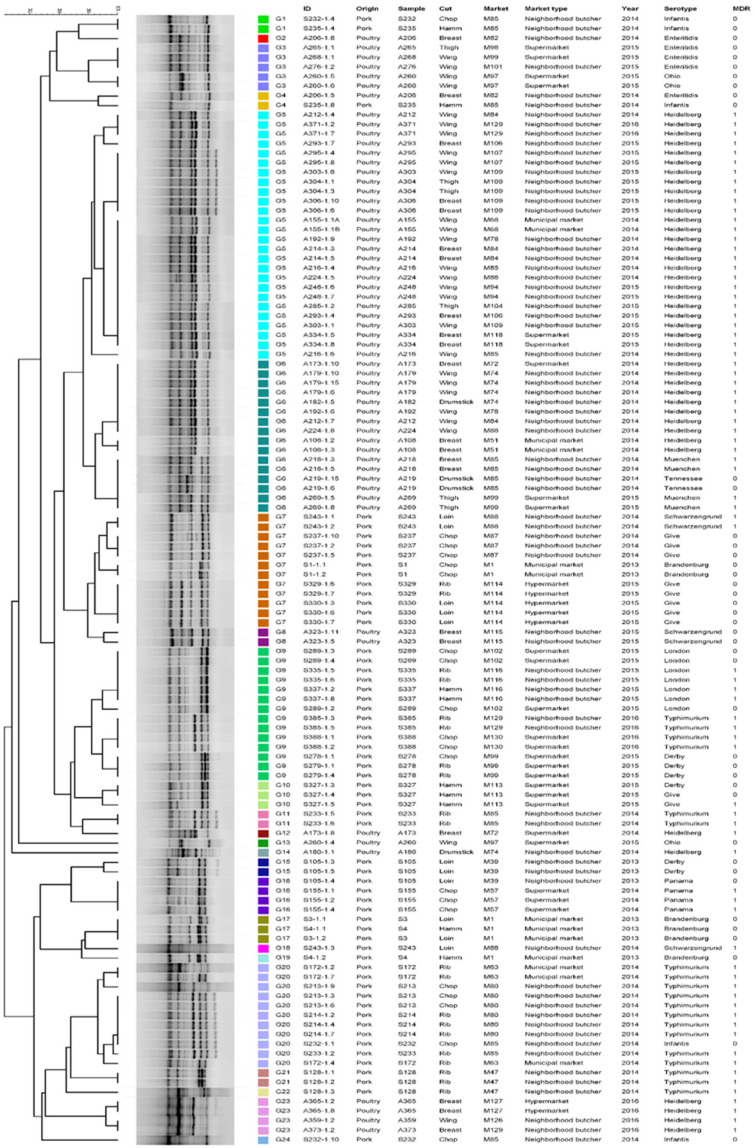
Dendrogram showing the relationship among the SE-AFLP genotypes of *S. enterica* isolates from pork and poultry meat.

The SE-AFLP analysis resulted in 24 clusters (G1–G24) (Figure 1). Most clusters are exclusively pork or poultry meat, except for G4, which comprises one isolate from pork and one from poultry meat that originated from distinct markets. There is a slight tendency to cluster isolates according to serotypes and markets; however, genotypes G7 and G9 stand out for clustering pork isolates from three distinct serotypes each.

**Figure 2:**
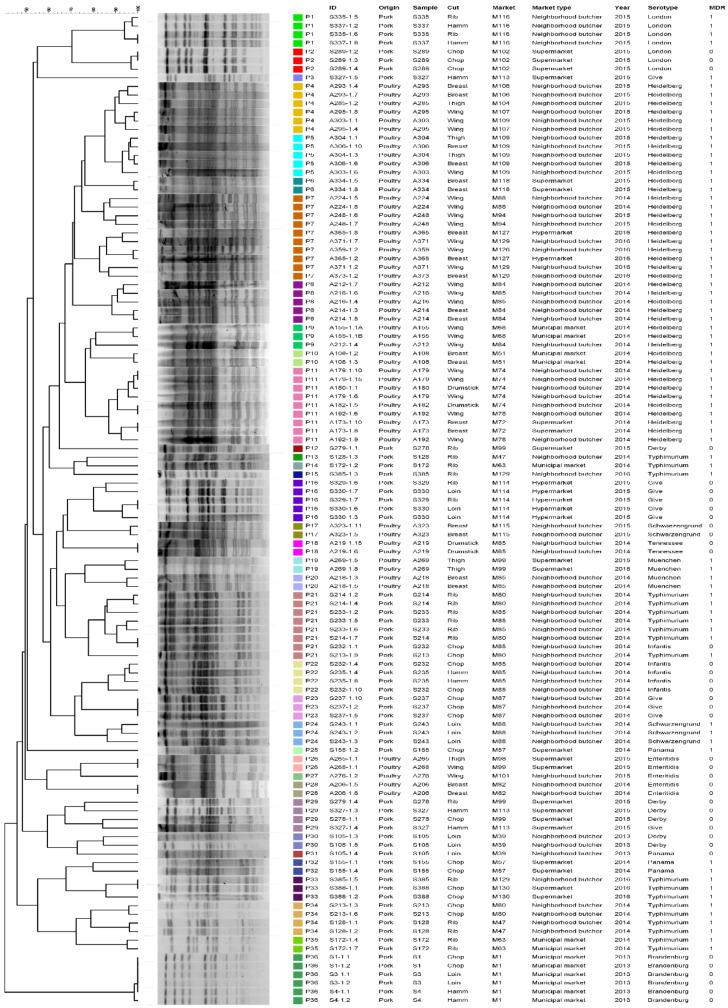
Dendrogram showing the relationship among the PFGE pulsotypes of *S. enterica* isolates from pork and poultry meat.

The PFGE analysis resulted in 36 pulsotypes (P1–P36) (Figure 2). Here, clusters are exclusively made up of pork or poultry meat, and clearly differentiate isolates according to their serotypes and markets. For both SE-AFLP and PFGE techniques, the isolates originating from the markets positive for both pork and poultry samples, simultaneously (M85, M88, M99, and M129), were also separated in distinct genotypes according to their serotypes. The discriminatory indexes for SE-AFLP and PFGE were 0.89 and 0.97, respectively.

The resistance rates of pork and poultry isolates against tested antimicrobials are presented in Table 3. There is a difference between the resistance profiles of pork and poultry isolates. While the pork isolates present higher resistance to azithromycin (95.0%) followed by ampicillin (51.7%), oxytetracycline (40.0%), chloramphenicol (40.0%), and nalidixic acid (38.3%), the poultry meat isolates stand out with higher resistance to quinolones (nalidixic acid and ciprofloxacin—82.8 and 74.1%, respectively), sulfamethoxazole (81.0%), oxytetracycline (79.3%), and over 69% resistance to tested β-lactams. Interestingly, colistin resistance was observed in only three poultry isolates (5.2%).

Multidrug resistance was detected in 50.0% of pork isolates and 79.3% of poultry isolates. Among *S. enterica* originated from pork samples, we highlight serotypes Typhimurium and Schwarzengrund with 100% of multi-resistant isolates, followed by Panama (75.0%) and London (57.1%) (Table 4). For the poultry isolates, serotypes Muenchen and especially Heidelberg stand out, with 100% multi-resistance, in which the serotype Heidelberg corresponds to 72.4% of the isolates of poultry origin.

**Figure 3:**
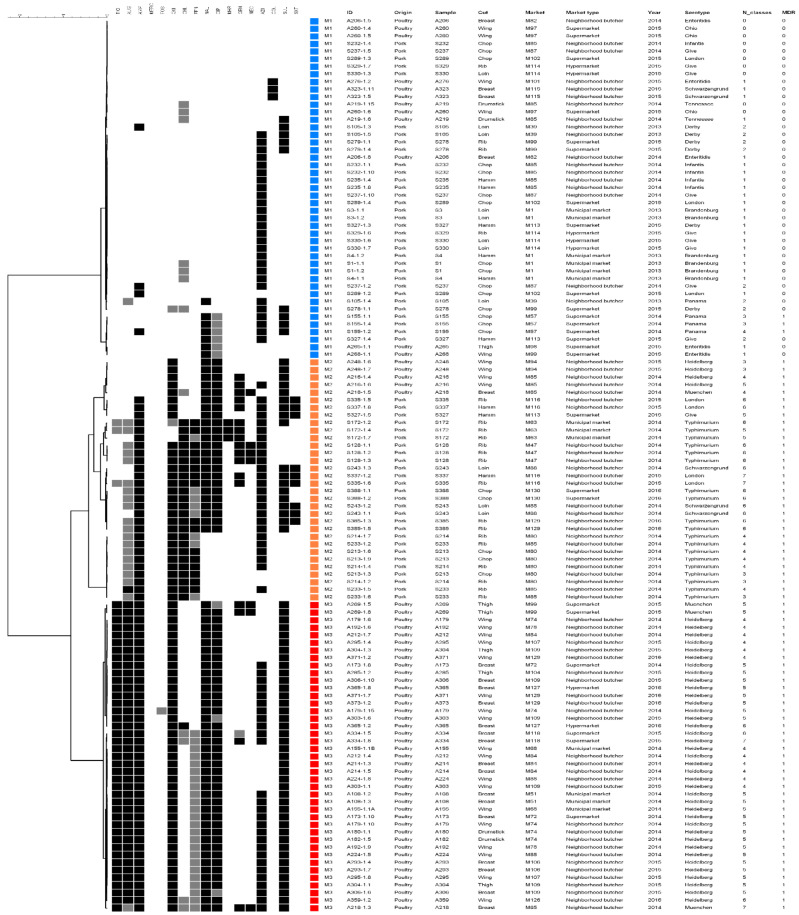
Antimicrobial resistance profiles cluster analysis of *S. enterica* isolates from pork and poultry meat. The grey scale squares (black, grey and white) correspond to resistant, intermediate and sensitive status, respectively.

The resistance profiles cluster analysis resulted in three clusters (M1–M3) (Figure 3). The M1 group corresponded to 45 isolates mostly of pork origin (73.3%), from 10 distinct serotypes, with only three multi-resistant Panama isolates. Interestingly, the three colistin resistant isolates of poultry origin were included in M1 as they only demonstrate resistance to this drug. The M2 cluster was composed of 32 multidrug resistant isolates, mostly from pork (84.4%), including all serotype Typhimurium isolates; these profiles assemble the isolates resistant to ampicillin, oxytetracycline and azithromycin, and with variable resistance to phenicols and quinolones. Finally, the M3 cluster comprised 41 multi-resistant poultry isolates, of which 92.7% were from serotype Heidelberg; these were resistant to β-lactams, oxytetracycline, quinolones and sulfamethoxazole.

## 4. Discussion

Despite the importance of salmonellosis to public health, data on *S. enterica* prevalence in retail meat products are still scarce in Brazil. Most research focuses on the production chain, especially slaughterhouses, in such a way that the risk of consumer exposure arising from products sold in markets is not fully known. The knowledge of *Salmonella* prevalence is necessary for estimating the risks of foodborne diseases related to meat consumption, and further characterization of isolates can be useful for understanding the origin of contamination and development of intervention strategies for risk reduction [28].

Here, we report a prevalence of 7.3% for *S. enterica* isolation from raw pork and poultry meat from retail markets in Brazil. The city of São Paulo, where this study was conducted, has a population of 12.2 million inhabitants, being the largest city in the Southern Hemisphere; it is considered a cosmopolitan city, home to citizens from all countries. Ristori et al. [28] recently reported 5.8% (32/552) of *S. enterica* prevalence in meat products sold at São Paulo retail, comprising mostly contaminated raw pork sausages and chicken legs. In contrast, Perin et al. [11] detected *Salmonella* in 31.7% of frozen chicken cuts (wing, breast, leg, and fried chicken) produced and commercialized in the state of Paraná, south Brazil. Mürmann et al. [29] also described an *S. enterica* isolation rate of 24.4% (82/672) in fresh pork sausages collected at retail level in 36 butcher’s shops and supermarkets in Porto Alegre, south Brazil. Variability of *Salmonella* prevalence in retail meat is commonly reported worldwide, ranging from 2.4% in Europe [30], 18.1% in Mexico [31], to over 35% in China and Cambodia [32,33,34].

Most of the *S. enterica* positive markets in this study were characterized as neighborhood butchers, which may be related to hygiene protocols and greater variability in the origin of meats offered for sale. Nevertheless, supermarkets and hypermarkets were also positive for *S. enterica* isolation (25.7% and 5.7% of positive markets, respectively) indicating that structural size and greatness of brands are not related to the absence of contamination. Usually, it is expected that supermarkets present a lower prevalence of *Salmonella* contamination than in traditional/wet markets due to the differences in hygiene conditions [32,35,36]. However, higher levels of *S. enterica* contamination in meat retailed at supermarkets have been reported in Mexico, as well as European and Asian countries, demanding attention to further understand this health threat [31].

Interestingly, in our study, most positive markets commercialized both pork and poultry meat; however, in only four markets was *S. enterica* isolated from both pork and poultry samples simultaneously, but interestingly, the isolates belonged to different serotypes. This suggests the absence of cross contamination between pork and poultry meat within markets and reinforces the possible origin of contamination from the production chain. This may be also sustained by genotyping results, in which both SE-AFLP and PFGE techniques resulted in most clusters exclusively of pork or poultry meat, and a tendency to differentiate isolates according to their serotypes and markets of origin.

In this study, we identified 12 different *S. enterica* serotypes, with a predominance of Heidelberg and Typhimurium in poultry meat and pork, respectively. High variability of serotypes among pork and poultry meat products had already been described in Brazil. Perin et al. [11] detected nine distinct serotypes among frozen chicken cuts and the majority was *S.* Typhimurium and *S.* Heidelberg. Similarly, Ristori et al. [28] reported 14 different serotypes in raw pork sausages and chicken legs, of which Typhimurium and Enteritidis were the most frequent among pork and poultry meat, respectively. Interestingly, in our study, only serotype Schwarzengrund appeared in both pork and poultry meat, and *S*. Infantis was restricted to pork samples. The serotype Schwarzengrund strains isolated from pork presented a multi-resistance profile (resistance to seven antimicrobial classes), very diverse of poultry strains of the same serotype, which were resistant only to colistin.

As expected, the prevalence of non-*S.* Enteritidis isolates from poultry meat refers to the change in the epidemiology of *Salmonella* in the country [37]. Serotypes Typhimurium and Heidelberg have been prevalent not only in meat but also among the poultry and porcine production chains [15,38,39]. These serotypes are also highlighted for presenting a multidrug resistant profile [11,39]; in our study, the totality of *S*. Typhimurium and *S*. Heidelberg were characterized as multidrug resistant. Moreover, serotypes London, Panama, Schwarzengrund, and Muenchen also presented over 50% of multidrug resistance.

Regarding the antimicrobial resistance, in addition to the difference between the resistance profiles of pork and poultry isolates, the high levels of resistance to a variety of antimicrobials demand further attention to both human and veterinary medicine. The traditional first-line antimicrobials for Salmonella infections were ampicillin, trimethoprim-sulfamethoxazole and chloramphenicol, and due to widespread resistance, currently, the use of fluoroquinolones, azithromycin, and extended-spectrum cephalosporin is recommended [5,40]. These are among the antimicrobials to which we observed higher resistance rates (Table 3) which demand further attention to the public health risks. Furthermore, the antimicrobials that we detected high resistance levels are also included in the WHO list [1] of CIA—critically important antimicrobials (azithromycin, 3rd generation cephalosporins, fluoroquinolones, ampicillin, and amoxicillin/clavulanate)—and HIA—highly important antimicrobials (trimethoprim-sulfamethoxazole, chlortetracycline, and chloramphenicol).

The resistance patterns observed between pork and poultry isolates are distinct and have a clear correlation with the selection pressure that has been carried out in recent years in different animal intensive production systems. While in swine there is a higher frequency of resistance to azithromycin (macrolide), ampicillin (β-lactam), and chloramphenicol (phenicol), which belong to classes widely used in Brazilian swine production [41], in poultry, there is relatively high resistance to quinolones, sulfamethoxazole, oxytetracycline, and β-lactams (ceftiofur, amoxicillin/clavulanate, and ampicillin), which are also extensively applied in poultry production systems [42].

Mellor et al. [43] compared the resistance profile in 3537 *S*. Typhimurium isolates isolated between 2003 and 2014 in the United Kingdom from swine, cattle, and chickens. The authors reported that the isolates of swine origin showed greater diversity of resistance profiles and higher multi-resistance rate when comparing with isolates of avian and bovine origin. In the present study, multi-resistant isolates were more frequent in poultry (79.3%) than in pork (50%). Interestingly, the Mellor et al. [43] ecological diversity analyses revealed variations in observed resistance profiles both between host species and between production types for chickens and pigs, similar to our results from meat isolates. The authors suggest that several factors, in addition to antimicrobial use, may influence the variation in *Salmonella* resistance profiles among host species, including host immunity, vaccination status, biosecurity, and industry structure.

## 5. Conclusions

Despite investments in good practices for farming and the meat industry, *Salmonella enterica* contamination remains a risk to human health. Monitoring the most important serovars in the final product and in animal production systems is of great importance so that the country can carry out applied control plans. Contamination was slightly higher in small butcher shops; nevertheless, supermarkets and hypermarkets also presented positive results and deserve attention. The observed resistance profiles and genotypes indicate that the meat contamination originates in the production systems or slaughterhouses and do not suggest cross contamination in the evaluated markets. High resistance rates to critically important antimicrobials for human health reinforces the importance of controlling and monitoring *Salmonella* contamination in these production chains.

## Figures and Tables

**Table 1:** Distribution of meat samples positive for *Salmonella enterica* isolation according to their origin.

City Region	Nº Positive Markets	Pork				Poultry				Total
		Chop	Rib	Loin	Hamm	Breast	Thigh	Drumstick	Wing	
Center	7/22	1/17	1/17	0/17	0/13	2/17	2/17	1/17	4/15	11/130
East	4/18	1/11	1/13	0/12	1/11	1/12	0/11	0/12	0/12	4/94
North	8/26	0/22	2/24	1/22	1/20	3/22	1/21	1/22	3/18	12/171
West	8/37	3/21	2/25	2/24	1/22	0/22	2/22	0/22	4/22	14/180
South	8/29	3/26	2/24	2/26	1/27	4/25	0/26	1/26	3/25	16/205
Total	35/132	8/97	8/103	5/101	4/93	10/98	5/97	3/99	14/92	57/780

**Table 2:** Distribution of serotypes detected among studied *Salmonella enterica* isolates—*n* (%).

Serotype	Pork	Poultry	Total
Heidelberg	-	42 (72.4)	42 (35.6)
Typhimurium	19 (31.7)	-	19 (16.1)
Give	10 (16.7)	-	10 (8.5)
London	7 (11.7)	-	7 (5.9)
Brandenburg	6 (10.0)	-	6 (5.1)
Derby	6 (10.0)	-	6 (5.1)
Enteritidis	-	5 (8.6)	5 (4.2)
Infantis	5 (8.3)	-	5 (4.2)
Schwarzengrund	3 (5.0)	2 (3.4)	5 (4.2)
Muenchen	-	4 (6.9)	4 (3.4)
Panama	4 (6.7)	-	4 (3.4)
Ohio	-	3 (5.2)	3 (2.5)
Tennessee	-	2 (3.4)	2 (1.7)
Total	60 (100)	58 (100)	118 (100)

**Table 3:** MIC range and resistance rates of *Salmonella enterica* isolates against tested antimicrobials.

Antimicrobial	Range (µg/mL)	Pork			Poultry		
		S N (%)	I N (%)	R * N (%)	S N (%)	I N (%)	R * N (%)
Ceftiofur	0.25–8	57 (95.0)	-	3 (5.0)	17 (29.3)	-	41 (70.7)
Amoxicillin/Clavulanate	1/0.5–32/64	39 (65.0)	20 (33.3)	1 (1.7)	17 (29.3)	1 (1.7)	40 (69.0)
Ampicillin	1–64	29 (48.3)	-	31 (51.7)	17 (29.3)	-	41 (70.7)
Meropenem	0.25–8	60 (100)	-	-	58 (100)	-	-
Fosfomycin	8–512	60 (100)	-	-	57 (98.3)	-	1 (1.7)
Oxytetracycline	2–32	34 (56.7)	2 (3.3)	24 (40.0)	12 (20.7)	-	46 (79.3)
Chloramphenicol	4–64	32 (53.3)	4 (6.7)	24 (40.0)	49 (84.5)	8 (13.8)	1 (1.7)
Florfenicol	0.5–8	36 (60.0)	8 (13.3)	16 (26.7)	34 (58.6)	-	24 (41.4)
Nalidixic Acid	8–128	37 (61.7)	-	23 (38.3)	10 (17.2)	-	48 (82.8)
Ciprofloxacin	0.06–8	38 (63.3)	4 (6.7)	18 (30.0)	10 (17.2)	5 (8.6)	43 (74.1)
Marbofloxacin	0.06–8	57 (95.0)	-	3 (5.0)	58 (100)	-	-
Gentamicin	0.5–32	50 (83.3)	-	10 (16.7)	50 (86.2)	1 (1.7)	7 (12.1)
Neomycin	4–16	57 (95.0)	-	3 (5.0)	54 (93.1)	-	4 (6.9)
Azithromycin	4–64	3 (5.0)	-	57 (95.0)	29 (50.0)	-	29 (50.0)
Colistin	1–16	60 (100)	-	-	55 (94.8)	-	3 (5.2)
Sulfamethoxazole	256–1024	39 (65.0)	-	21 (35.0)	11 (19.0)	-	47 (81.0)
Trimethoprim/Sulfamethoxazole	2/18–4/76	51 (85.0)	-	9 (15.0)	58 (100)	-	-

* Gray cells highlight the antimicrobials with the highest resistance rate in poultry and pork.

**Table 4:** Number of resistant antimicrobial classes according to serotypes detected among pork and poultry *S. enterica* isolates—*n* (%).

Origin	Serotype	Nº Resistant Antimicrobial Classes			Total
		0–2	3–5	>6	
Pork	Typhimurium	-	11 (57.9)	8 (42.1)	19 (100)
	Give	9 (90.0)	1 (10.0)	-	10 (100)
	London	3 (42.9)	-	4 (57.1)	7 (100)
	Brandenburg	6 (100)	-	-	6 (100)
	Derby	6 (100)	-	-	6 (100)
	Infantis	5 (100)	-	-	5 (100)
	Panama	1 (25.0)	3 (75.0)	-	4 (100)
	Schwarzengrund	-	-	3 (100)	3 (100)
	Total	30 (50.0)	15 (25.0)	15 (25.0)	60 (100)
Poultry	Heidelberg	-	38 (90.5)	4 (9.5)	42 (100)
	Enteritidis	5 (100)	-	-	5 (100)
	Muenchen	-	3 (75.0)	1 (25.0)	4 (100)
	Ohio	3 (100)	-	-	3 (100)
	Schwarzengrund	2 (100)	-	-	2 (100)
	Tennessee	2 (100)	-	-	2 (100)
	Total	12 (20.7)	41 (70.7)	5 (8.6)	58 (100)

## Data Availability

The data that support the findings of this study are available from the corresponding author (AMM), upon reasonable request.

## Supplementary Materials

The following supporting information can be downloaded at: https://www.mdpi.com/article/10.3390/pathogens11030358/s1, Table S1. Distribution of serotypes detected among studied *Salmonella enterica* isolates according to their origin.
