# Supplementary material for: Characterization of Salmonella enterica Contamination in Pork and Poultry Meat from São Paulo/Brazil: Serotypes, Genotypes and Antimicrobial Resistance Profiles
Source: Pathogens. 2022 Mar 16;11(3):358. doi: 10.3390/pathogens11030358 (PMC8951033; doi:10.3390/pathogens11030358)
Supplement: Supplementary file 1 [file pathogens-11-00358-s001.zip › pathogens-1602596-supplementary.pdf]

Table S1: Distribution of serotypes detected among studied *Salmonella enterica* isolates according to their origin.

Market	Meat Cut	Brandenburg	Derby	Enteritidis	Give	Heidelberg	Infantis	London	Muenchen	Ohio	Panama	Schwaezengrund	Tennessee	Typhimurium	Total
**M1**	Chop	2													2
	Hamm	2													2
	Loin	2													2
**M39**	Loin		2								1				3
**M47**	Rib													3	3
**M51**	Breast					2									2
**M57**	Chop										3				3
**M63**	Rib													3	3
**M68**	Wing					2									2
**M72**	Breast					2									2
**M74**	Drumstick					2									2
	Wing					3									3
**M78**	Wing					2									2
**M80**	Chop													3	3
	Rib													3	3
**M82**	Breast			2											2
**M84**	Breast					2									2
	Wing					2									2
**M85**	Breast								2						2
	Drumstick												2		2
	Wing					2									2
	Chop						3								3
	Hamm						2								2
	Rib													3	3
**M87**	Chop				3										3
**M88**	Loin											3			3
	Wing					2									2

**M94**	Wing					2									2
**M97**	Wing									3					3
**M98**	Thigh			1											1
**M99**	Chop		1												1
	Rib		2												2
	Thigh								2						2
	Wing			1											1
**M101**	Wing			1											1
**M102**	Chop							3							3
**M104**	Thigh					1									1
**M106**	Breast					2									2
**M107**	Wing					2									2
**M109**	Breast					2									2
	Thigh					2									2
	Wing					2									2
**M113**	Hamm		1		2										3
**M114**	Loin				3										3
	Rib				2										2
**M115**	Breast											1			2
**M116**	Hamm							2							2
	Rib							2							2
**M118**	Breast					2									2
**M126**	Wing					1									1
**M127**	Breast					2									2
**M129**	Breast					1									1
	Wing					2									2
	Rib													2	2
**M130**	Chop													2	2
**Total**		6	6	5	10	42	5	7	4	3	4	4	2	19	118
